# Supplementary material for: The incidence rate and influence factors of hemolysis, lipemia, icterus in fasting serum biochemistry specimens
Source: PLoS One. 2022 Jan 19;17(1):e0262748. doi: 10.1371/journal.pone.0262748 (PMC8769349; doi:10.1371/journal.pone.0262748)
Supplement: Attachment — Submitted filename: Revisions HIL.pdf [file pone.0262748.s002.pdf]

The incidence rate of hemolysis, lipemia, icterus and influence factors: A single-center retrospective study of 501,612 fasting serum biochemistry specimens

Comment: The title should be revised, there is no need to indicate the sample size. Influencing factors may not fit based on the reasons that authors will find in major comments.

Major revisions: About Study design:

Authors should emphasize that this a descriptive study in their methodology . The authors indicated that the objective was to evaluate and link pre analytical factors like diet, drugs, collecting, handling, transportation, and preparing specimens. Yet, in their results, they are not interpreted and related to the HIL. Again, there are no methodology that highlight or show how these factors were collected.

- So, authors should refine their topic as well as their objective and as well as their research question
- Authors should refine their study and emphasize on gender and department according to their results presentation and these the tangible data collected according to their retrospective studies
- More of the findings, should be discussed generally and maybe recommended

In discussion part:

Authors should discuss specifically on the effects of the HIL for the lab results according to each parameter. For eg. physiological lipemia may not a wide number of tests being requested. Hemolysis may affect only hematology and maybe Potassium ion results but not others.

In conclusion part: Authors said they studied influence of factors of HIL, I suggest they should remove these factors as they were not investigated, rather they assumed theoretically as seen in their discussion.

Authors should rather emphasize on incidence rate as the topic highlights.
