# Supplementary material for: The incidence rate and influence factors of hemolysis, lipemia, icterus in fasting serum biochemistry specimens
Source: PLoS One. 2022 Jan 19;17(1):e0262748. doi: 10.1371/journal.pone.0262748 (PMC8769349; doi:10.1371/journal.pone.0262748)
Supplement: Attachment — Submitted filename: Respond to Reviewers.docx [file pone.0262748.s003.docx]

**Reviewer #1:**

Dear Authors,
Thank you for the interesting manuscript. My recommendations have been listed below for this manuscript:

1. Page 4: "The HIL index is defined as the lowest concentrations of HIL that interfere with chemical analyses, yielding a bias >10% based on the Clinical and Laboratory Standards Institute (CLSI) document C56-A (CLSI-C56-A)." In the CLSI document, an allowable bias of 10% is just presented as an example and cannot be generalized. Therefore, this sentence should be rewritten.

**Response:** Thank you for spending your valuable time reading the manuscript and giving insightful suggestions to help us improve the quality of our manuscript. According to your suggestion, we carefully checked this sentence and rewrote it in the revised manuscript.
2. Page 6: "We finally included 501,612 eligible fasting serum biochemical specimens for analysis." Here, please explain how the fasting information of the patients had been obtained.

**Response:** Thanks for these insightful suggestions. The study investigated the incidence rate of hemolysis, lipemia, icterus, and influence factors based on fasting serum biochemistry specimens. Therefore, we used SIEMENS ADVIA 2400 biochemical analyzers to perform routine biochemical index tests, including proteins, enzymes, metabolites, fasting and postprandial glucose, blood lipids, and electrolytes. In order to decrease the influence of diet, we only included specimens labeled fasting blood samples (verification by blood collection nurses) by searching the LIS records retrospectively. Besides, HIL data were further excluded if the sampling time was beyond the fasting blood collection period (6:00 am to 11:30 am). Finally, the authors and seven medical college students separately checked each record to exclude potential postprandial specimens, especially postprandial glucose, pancreatitis markers (e.g., amylase, lipase, and amylopsin), and plasma specimens. In general, we took more than eleven months to check patients' data and information, including gender, age, departments transportation time, and the detection time of specimens. Though we try our best to decrease the influence of diet for the measurements of routine serum biochemical specimens, it is hard to ensure each specimen is fasting. Therefore, we added these limitations in the discussion of the revised manuscript.
3. Page 17: "A previous study found that longer transit time increased the risk of hemolysis [27]. Meanwhile, it is crucial to reduce turnaround time for early diagnosis and treatment by providing the safety and pleasure of patients [26]." Please add own results and discuss them.

**Response:** Thanks for your valuable advance. We added our results and discussed them.
4. Page 17-18: "Besides, another study also indicated that in vivo aging of red blood cells is associated with increased cellular density, corresponding to increased cell age [28]. These findings may, in part, explain the increased tendency of hemolysis in adults aged more than 40." In my opinion, here, the increasing cell age have been confused with the patient age. It should be reconsidered.

**Response:** Thanks for your valuable suggestion. We carefully checked these sentences and modified them in the revised manuscript.
5. Page 19: "Our data revealed that the most common suspected causes of the markedly elevated lipemic index were lipid emulsion (Gastrointestinal Surgery) and hemolysis (Neonatology Department), consistent with these previous reports." Here, please give information about the patients treated with lipid emulsion. Otherwise, this statement should be reconsider.

**Response:** Thanks for your valuable suggestion. We carefully checked the electronic medical records of patients hospitalized in Gastrointestinal Surgery and modified them in the revised manuscript.
6. Page 20: "creatine" or "creatinine"?

**Response:** I am very sorry for these mistakes. We carefully checked the whole manuscript to avoid any linguistic or spelling errors.
7. The "hemolysis in vitro" should be changed to "in vitro hemolysis" thoroughly the manuscript.

**Response:** We changed "hemolysis in vitro" to "in vitro hemolysis" according to your suggestion.
8. Discussion have been written as if a book chapter. It should be shortened to the purpose of the study.

**Response:** Thanks for your valuable suggestion. We rewrote the discussion in the revised manuscript.
9. The style of the references should be changed according to the requirements of the journal.

**Response:** Thanks for your suggestion. The style of the references has been changed according to the requirements of the journal.

**Reviewer #2:**

The incidence rate of hemolysis, lipemia，icterus and influence factors: A single-center retrospective study of 501,612 fasting serum biochemistry specimens

Comment: The title should be revised, there is no need to indicate the sample size. Influencing factors may not fit based on the reasons that authors will find in major comments.
**Response:** We would like to thank you for the appreciation of our submitted manuscript and thank you once more for taking the time and effort to provide these very constructive and insightful suggestions. According to your suggestion, we revised the whole manuscript, revised the title, and rewrote the whole manuscript.
Major revisions: About Study design:
Authors should emphasize that this a descriptive study in their methodology . The authors indicated that the objective was to evaluate and link pre analytical factors like diet, drugs, collecting, handling, transportation, and preparing specimens. Yet, in their results, they are not interpreted and related to the HIL. Again, there are no methodology that highlight or show how these factors were collected.
• So, authors should refine their topic as well as their objective and as well as their research question.

**Response:** We modified the whole manuscript, emphasized that this is a descriptive study in methodology, and refined the topic, objective, and research questions as you suggested.
• Authors should refine their study and emphasize on gender and department according to their results presentation and these the tangible data collected according to their retrospective studies

**Response:** Thanks for your valuable suggestion. We refined the study and emphasized gender and department according to the actual data collected.
• More of the findings, should be discussed generally and maybe recommended
In discussion part:
Authors should discuss specifically on the effects of the HIL for the lab results according to each parameter. For eg. physiological lipemia may not a wide number of tests being requested. Hemolysis may affect only hematology and maybe Potassium ion results but not others.

**Response:** We rewrote the discussion in the revised manuscript.
In conclusion part: Authors said they studied influence of factors of HIL, I suggest they should remove these factors as they were not investigated, rather they assumed theoretically as seen in their discussion.

**Response:** According to your suggestion, we removed not investigated factors.
Authors should rather emphasize on incidence rate as the topic highlights.

**Response:** Many thanks for your insightful suggestion. We also emphasized the incidence rate as the topic highlights.
